# Supplementary material for: A Predictive Pharmacokinetic Model for Immune Cell-Mediated Uptake and Retention of Nanoparticles in Tumors
Source: Int J Mol Sci. 2022 Dec 10;23(24):15664. doi: 10.3390/ijms232415664 (PMC9779081; doi:10.3390/ijms232415664)
Supplement: Supplementary file 1 [file ijms-23-15664-s001.zip › ijms-2005379-supplementary.pdf]

Supplementary Information

Article

A Predictive Pharmacokinetic Model for Immune Cell-Mediated Uptake and Retention of Nanoparticles in Tumors

Ailton Sousa-Junior [1,2,†], Chun-Ting Yang [3,†], Preethi Korangath [3], Robert Ivkov [3,4,5,6,*] and Andris Bakuzis [1,7,*]

[1] Instituto de Física, Universidade Federal de Goiás, Goiânia 74690-900, GO, Brazil
[2] FarmaTec—Laboratório de Tecnologia Farmacêutica, Universidade Federal de Goiás, Goiânia 74690-631, GO, Brazil
[3] Department of Radiation Oncology and Molecular Radiation Sciences, Johns Hopkins University School of Medicine, Baltimore, MD 21218, USA
[4] Department of Oncology, Johns Hopkins University School of Medicine, Baltimore, MD 21218, USA
[5] Department of Mechanical Engineering, Whiting School of Engineering, Johns Hopkins University, Baltimore, MD 21218, USA
[6] Department of Materials Science and Engineering, Whiting School of Engineering, Johns Hopkins University, Baltimore, MD 21218, USA; rivkov1@jhmi.edu
[7] CNanoMed, Universidade Federal de Goiás, Goiânia 74690-631, GO, Brazil
* Correspondence: rivkov1@jhmi.edu (R.I.); bakuzis@ufg.br (A.B.)
† These authors contributed equally to this work.

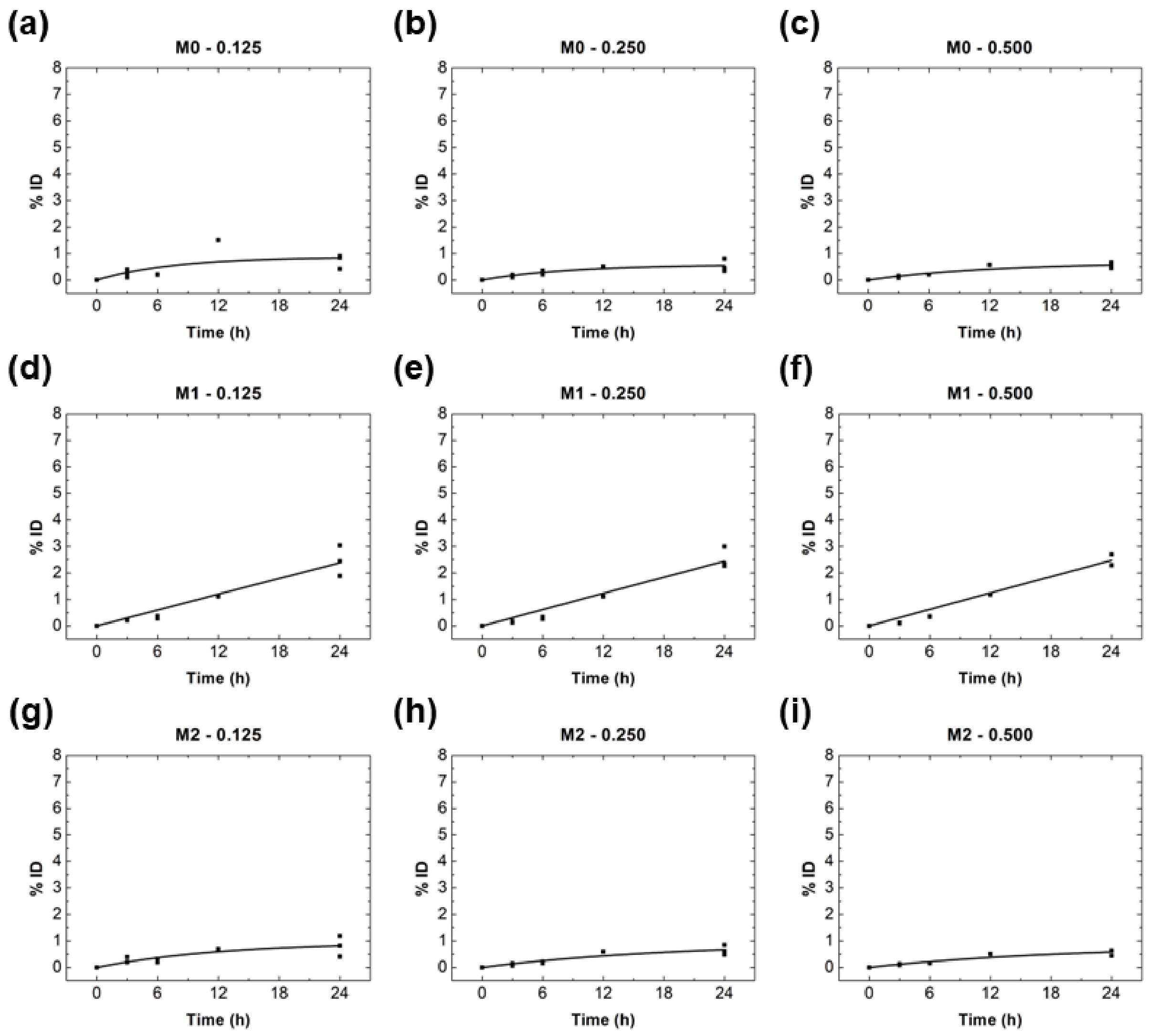

Figure S1. *In vitro* BNF-Plain uptake by macrophages. Fitting of the macrophage internalization kinetics $x_{in}(t)$ (Equation 17 in the manuscript) using all *in vitro* experimental values obtained for BNF-Plain (ferene-s assay at 3, 6, 12, and 24 h, replicates included), for three different initial doses (0.125, 0.250, or 0.500 mg of Fe) and three macrophage polarization states (M0, M1, and M2).

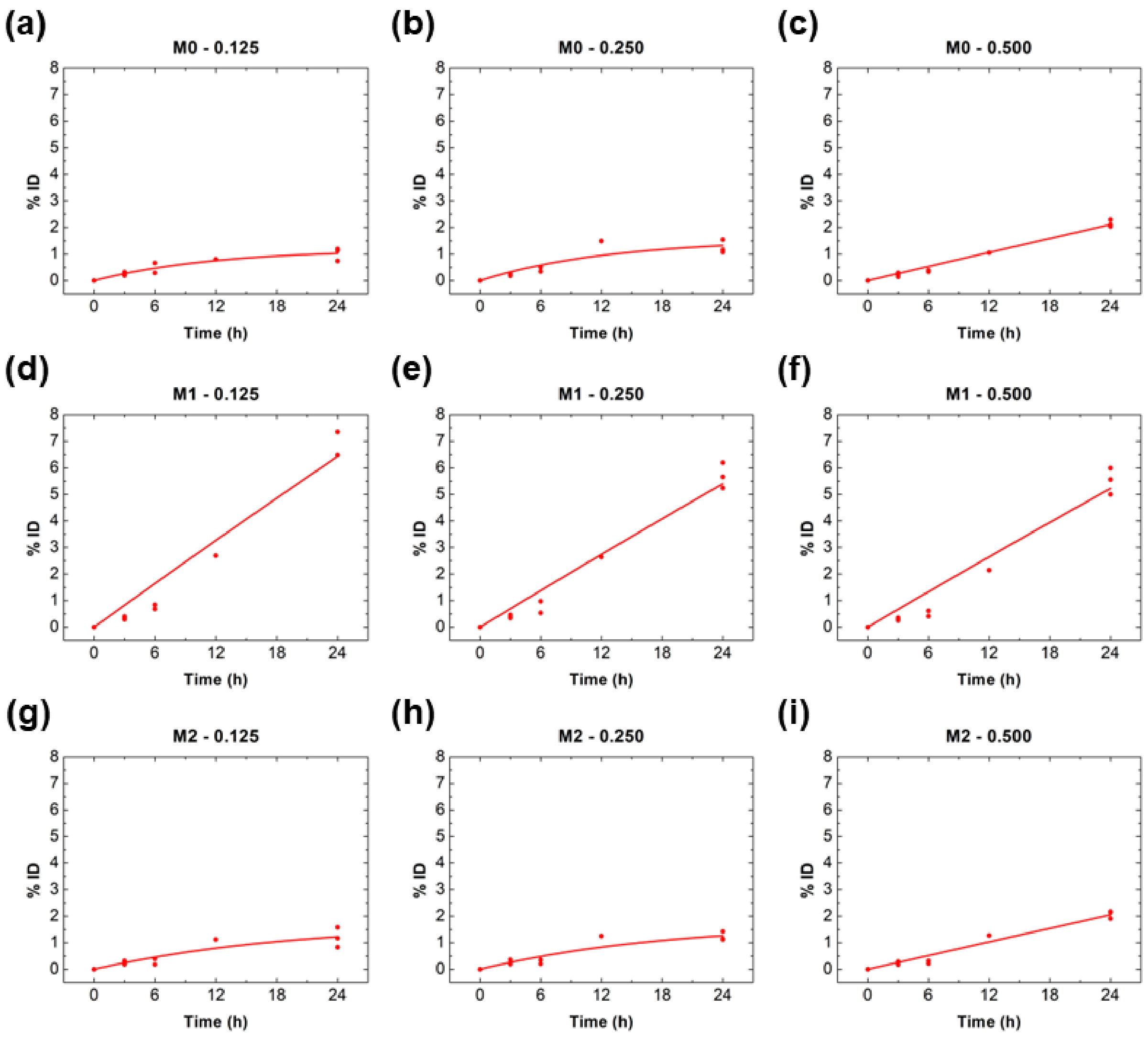

Figure S2. *In vitro* BNF-Her uptake by macrophages. Fitting of macrophage internalization kinetics $x_{in}(t)$ (Equation 17 in the manuscript) using all *in vitro* experimental values obtained for BNF-Her (ferene-s assay at 3, 6, 12, and 24 h, replicates included), for three different initial doses (0.125, 0.250, or 0.500 mg of Fe) and three macrophage polarization states (M0, M1, and M2).

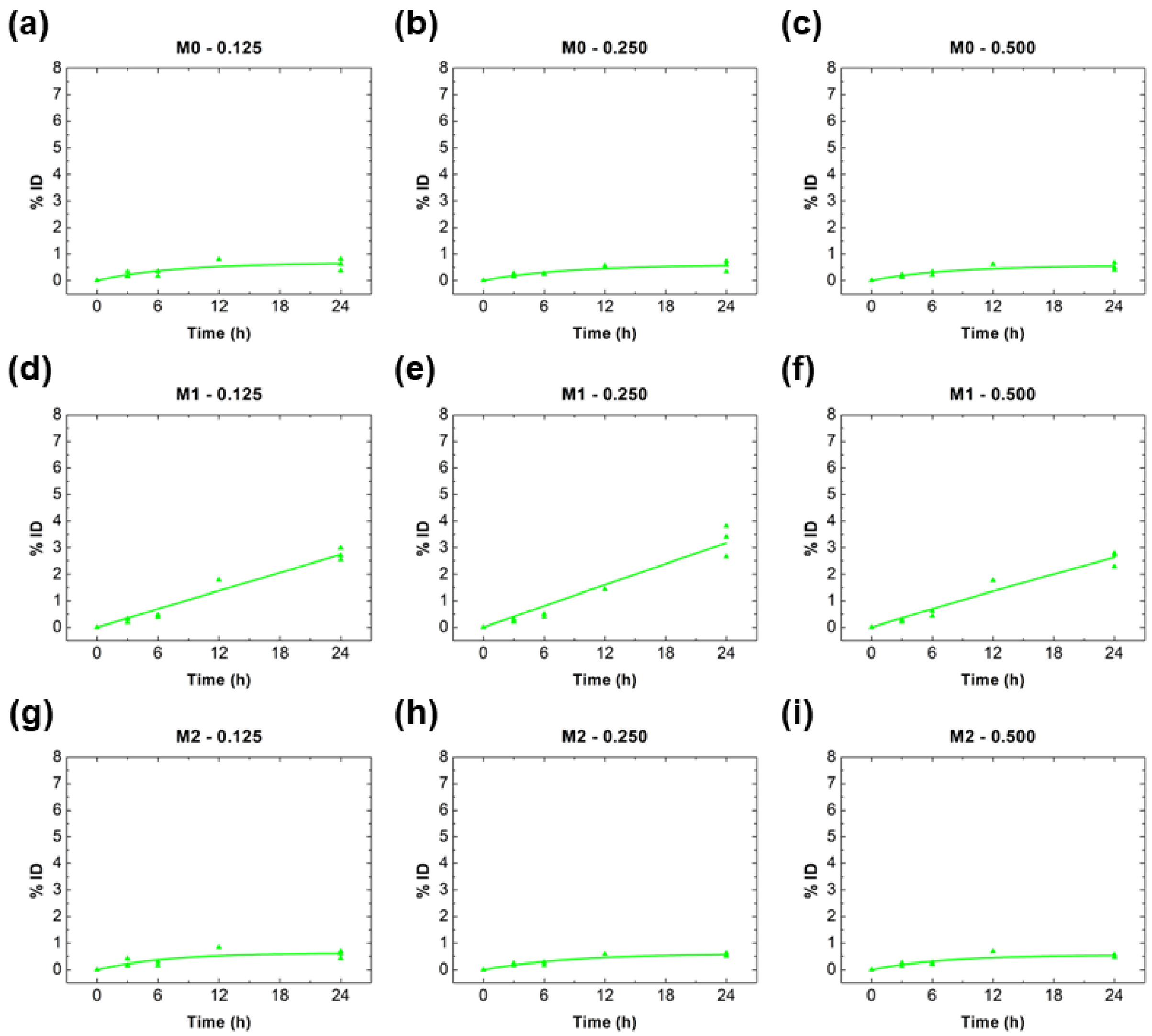

Figure S3. *In vitro* BNF-IgG uptake by macrophages. Fitting of macrophage internalization kinetics $x_{in}(t)$ (Equation 17 in the manuscript) for all *in vitro* experimental values obtained for BNF-IgG (ferene-s assay at 3, 6, 12, and 24 h, replicates included), for three different initial doses (0.125, 0.250, or 0.500 mg of Fe) and three macrophage polarization states (M0, M1, and M2).

Table S1. Values of K_{in} and K_{out} obtained from the fitting shown in Figures S1, S2, and S3 (fittings with all datapoints, including replicates, for each experimental setup)

BNF-Plain					
		0.125	0.250	0.500	Average*
M0	K_{in}	0,00114 ± 0,00072	0,00065 ± 0,00024	0,00052 ± 0,00014	0,00077 ± 0,00026
	K_{out}	0,12955 ± 0,11133	0,11112 ± 0,05940	0,07854 ± 0,03447	0,10640 ± 0,04360
M1	K_{in}	0,00100 ± 0,00034	0,00103 ± 0,00029	0,00104 ± 0,00023	0,00102 ± 0,00017
	K_{out}	0,00000 ± 0,02931	0,00000 ± 0,02459	0,00000 ± 0,01951	0,00000 ± 0,01432
M2	K_{in}	0,00077 ± 0,00034	0,00049 ± 0,00016	0,00042 ± 0,00010	0,00056 ± 0,00013
	K_{out}	0,07987 ± 0,05917	0,05260 ± 0,03734	0,05238 ± 0,02794	0,06162 ± 0,02511

BNF-Her					
		0.125	0.250	0.500	Average*
M0	K_{in}	0,00100 ± 0,00025	0,00121 ± 0,00039	0,00089 ± 0,00012	0,00103 ± 0,00016
	K_{out}	0,08307 ± 0,03440	0,07694 ± 0,04265	0,00000 ± 0,01229	0,05334 ± 0,01872
M1	K_{in}	0,00277 ± 0,00068	0,00231 ± 0,00050	0,00223 ± 0,00061	0,00244 ± 0,00035
	K_{out}	0,00000 ± 0,02137	0,00000 ± 0,01884	0,00000 ± 0,02373	0,00000 ± 0,01236
M2	K_{in}	0,00089 ± 0,00033	0,00095 ± 0,00028	0,00086 ± 0,00018	0,00090 ± 0,00016
	K_{out}	0,05085 ± 0,04213	0,05385 ± 0,03495	0,00000 ± 0,01832	0,03490 ± 0,01924

BNF-IgG					
		0.125	0.250	0.500	Average*
M0	K_{in}	0,00089 ± 0,00034	0,00072 ± 0,00022	0,00075 ± 0,00021	0,00078 ± 0,00015
	K_{out}	0,13067 ± 0,06873	0,11783 ± 0,05018	0,12914 ± 0,04866	0,12588 ± 0,03268
M1	K_{in}	0,00116 ± 0,00025	0,00134 ± 0,00038	0,00117 ± 0,00025	0,00122 ± 0,00017
	K_{out}	0,00000 ± 0,01879	0,00000 ± 0,02460	0,00450 ± 0,01867	0,00150 ± 0,01205
M2	K_{in}	0,00091 ± 0,00036	0,00068 ± 0,00014	0,00076 ± 0,00022	0,00078 ± 0,00015
	K_{out}	0,14298 ± 0,07659	0,11205 ± 0,03244	0,13642 ± 0,05334	0,13048 ± 0,03294

*Average of the values found for each initial dose (0.125, 0.250, and 0.500 mg of Fe).

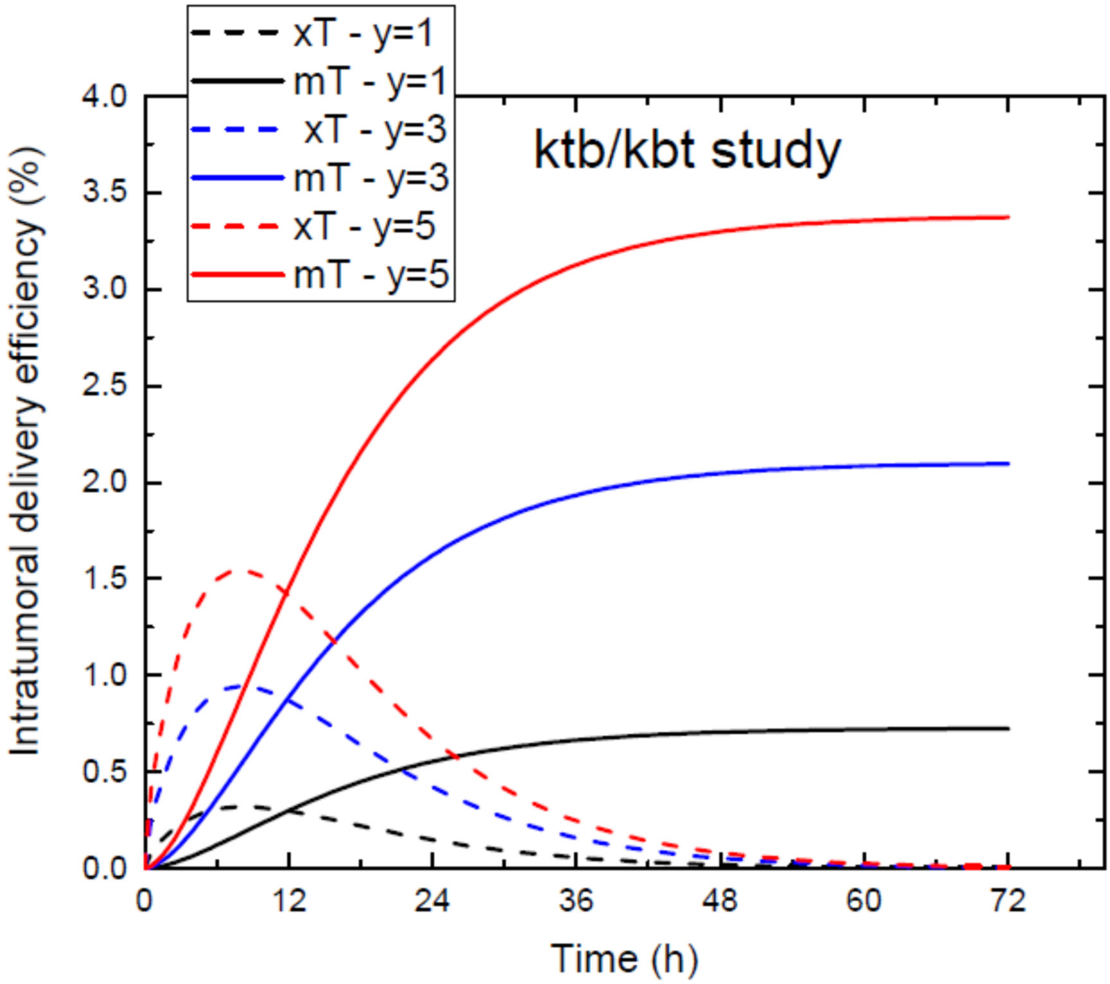

Figure S4. Simulated intratumor retention of BNF-Her nanoparticles. Simulated tumor retention of 100-nm BNF-Her nanoparticles varying the parameter K_{bt} and K_{tb} by increasing a factor y. Dashed lines represent passive retention (x_i), and solid-colored lines represent active retention by macrophages (m_i).
